# Detection of Delamination in Polymer Composites by Digital Image Correlation—Experimental Test

**DOI:** 10.3390/polym11030523

**Published:** 2019-03-20

**Authors:** Gábor Szebényi, Viktor Hliva

**Affiliations:** Department of Polymer Engineering, Faculty of Mechanical Engineering, Budapest University of Technology and Economics, H-1111 Budapest, Hungary; hlivav@pt.bme.hu

**Keywords:** delamination, digital image correlation, non-destructive test

## Abstract

Fiber-reinforced polymer composite structures are frequently used in industries where personal safety is critical; therefore, it is important to periodically estimate or monitor the condition of high value, load bearing structures. The digital image correlation (DIC) is well known as an effective method to obtain full field surface strains; in this paper, it was used to detect artificial damage inside the structures. Carbon or glass fabric reinforced epoxy specimens were produced and tested. All specimens contained an artificial through-delamination which was created by the insertion of different foils of a mould release agent during production. Tensile and compression tests were done while the camera system collected the images of the deformed surface to be analyzed posteriorly. In most cases the approximate locations of delaminations could be effectively detected from strain maps by the localization of zones showing different strain values than intact zones.

## 1. Introduction

In the 21st century, fiber-reinforced polymer composite structures are frequently used due to their excellent specific mechanical properties. With their use, we are able to build lighter structures and more efficient machines. The main applications of composites are the aerospace and automotive industry, wind turbines, pressure vessels and sport equipment. These applications are critical in terms of personal safety; therefore, it is important to periodically estimate or monitor the condition of high value, load-bearing structures. The situation is aggravated by the non-prognostic catastrophic failure of composites. Structural failure is caused in most cases by the synergy of multiple failure processes: fiber breakage, matrix fracture, fiber-matrix debonding/pullout and delamination which arise during use or even production [1,2,3].

Initially, detection of imperfections in composites was a more complicated task because it is made of two or more constituent materials with significantly different physical properties, quite different than the previously used quasi homogeneous metals. Nowadays it is not a problem thanks to the technical and computer processing capacity developments of the last 30 years. There are many non-destructive tests used by the industry for detecting imperfections including, but not limited to, computer tomography (CT), infrared thermography (IRT), acoustic emission (AE) and ultrasound test (UT). The topic has been investigated by several research groups but the perfect, non-destructive method that effectively detects all structural failure in any situation has not been found yet. For example, CT needs a stationary and very expensive machine and the geometry of the specimen is limited. Thermography needs a large energy input and a longer measurement time to show the internal damage in composite laminates. The AE method can only compare the recorded data to previously acquired references, and it needs the cracks to propagate during testing. UT needs contact between the specimen and the testing equipment by coupling gel; it is very time-consuming, and the measurement is very sensitive to machine setup [4].

In this work the digital image correlation (DIC) method is used, which is an old [5] but powerful tool for obtaining displacement and strains field during the test. The basic experimental setup consists of a digital camera which records the deformation of the loaded specimen in a material tester, a computer with DIC software and last, but not least, high-performance illumination. The way of software operation is that if one point is selected in the reference image it can be found in the next images thanks to a window which is around the point. Depending on the size, the window consists of some monochrome pixels which grant unique light intensity distribution (grey characteristic) to the window. The next image is scanned by a cross correlation method to detect the area with the most similar intensity distribution, which contains the required point. Thus, the movement of the point can be followed. If movements of two separate points are followed by DIC, it can compute the change of distance between the points. This can be used to exactly determine the elongation in a material test. Furthermore, if many points are examined then the full-field strain can be obtained. Of course, all of this only works if the examined specimen has a unique pattern with proper contrast on the surface of the specimen. It is a non-contact method, so all materials can be examined [6].

DIC techniques can be classified into three methods. Using one camera, the 2D DIC method can follow changes only in a plane. It is useful for obtaining transversal and longitudinal elongations during tensile or compression test [7,8]. The propagation or growing of cracks also can be followed in 2D during an interlaminar test. Determination of material properties (elastic modulus, Poisson’s ratio) is often important, for example, for finite element simulation [9]. Additionally, analyzing the full field strain can help understand the mode of failure. It is possible to process images afterwards, even SEM (scanning electron microscopy), AFM (atomic force microscopy), etc. images can be imported into the processing software [6]. In the real situation, the changes are mostly happening in 3D. When the deformation cannot be examined in a 2D plane or more accuracy is needed, two cameras are used for the 3D DIC technique [10,11,12,13]. However, it demands more complex calculation. If CT or MRI (magnetic resonance imaging) 3D data are available, they can be evaluated by volumetric digital image correlation (VDIC). This provides an opportunity to examine changes in an internal cavity or crack. 

These methods are able to detect structural faults, because under load the deformation of an intact object is different than the deformation of an imperfect object. If the fault of the structure is significant enough, the effect of damage may appear in a surface strain map. This phenomenon and the corresponding non-destructive methods are researched by many academics and industrial experts worldwide.

Chen et al. [14] analyzed the full field strain map of damaged composite plates during tensile and compression test by DIC. They found areas with pronounced strain values which marked the place of the barely visible structural faults. During the tensile test the strain aberration was positive around the damage, which was caused by fiber cracks. Under compression, some speckles will generate squash, buckling and even light reflection due to compressive deformation. The grey characteristic changes considerably compared to the original grey scale, which resulted in a relatively noisy strain map. The strain aberration was caused by local buckling. The effect of different parameters on the results was also showed. 

Sztefek and Olsson [15] analyzed low velocity impact-damaged carbon/epoxy composite plates by DIC. They have developed the inverse method which was used to generate nonlinear stress-strain curves of the damages zone. During the test, the plates’ deviation from flatness was determined on both sides, which allowed tracking of the opening of the delamination. Moreover, the local buckled zones were imported to the FE model, which results in a more accurate simulation.

Devivier et al. [16] examined the correlation between full-field measurements and numerical simulation results for multiple composite cantilever beams which contained artificial delamination. They identified and located the delamination by deflectometry from 2 strain components, which showed good correlation too. They noted that DIC requires two differentiations to obtain the strains; therefore, the noise will be strongly emphasized, in contrast with deflectometry, which requires only one differentiation and is insensitive to vibrations.

Wang et al. examined composite plates with and without defects by amplitude-fluctuation (AF) and electronic speckle pattern interferometry (ESPI) methods. The square- and circular-shape defects were created by PTFE foil placed between the prepreg layers. Global and local effects of the defect were shown on the resulting anomaly of the AF-ESPI fringe patterns at higher frequencies.

Like the previous examples, our aim is to find the connection between the surface deformation field and the damage inside the structure at small loads. In this article, delamination, as one of the major damage modes of composites, is examined. The structural defects are artificially created in various ways and they are tested by 2D DIC.

## 2. Materials and Methods

### 2.1. Production of Specimens

Three composite plates (I, II, III) were produced by hand lay-up followed by vacuum pressing for 2 h in 0.5 bar vacuum at room temperature. The matrix was IPOX MR 3012 (IPOX Chemicals, Budapest, Hungary) aliphatic epoxy resin (viscosity: 100–200 mPa∙s at 25 °C, epoxy equivalent: 0.65–0.75 g/equiv) with IPOX MH 3124 amine-based curing agent (viscosity: 80–120 mPa∙s at 25 °C, amine value: 464–490 mg KOH/g). The mixing weight ratio of the resin and the curing agent was 100:40, according to the producer’s specifications. Furthermore 1m% black coloring paste (Novia kft., Budapest, Hungary) was used.

As reinforcement, Sigratex C W200-PL1/1 (SGL Technologies Gmbh., Meitingen, Germany) carbon fabric (surface weight 200 g/m^2^) was used for the type I and II plates. E 220 (Saint-Gobain Vertex s.r.o., Litomysl, Czech Republic) glass fabric (surface weight 220 g/m^2^) was used for type III plate. 

The imperfection in the type I plate was generated by a 0.2 mm thick and 30 mm wide polytetrafluoroethylene (PTFE) foil strip (produced by PEMÜ Zrt., Solymár, Hungary) laminated between the reinforcing layers. [0/90^f^, ±45^f^, i, ±45^f^, 0/90^f^] stacking sequence was used, where *i* indicates the location of the PTFE foil. Imperfection of the type II plate was generated by Formula Five Mold Release wax (Rexco, Conyers, USA), which was applied directly to the middle layer in a 30 mm wide stripe. [0/90^f^, ±45^f^, *i*, ±45^f^, 0/90^f^] stacking sequence was used, where *i* indicates the location of the imperfection. The imperfection of the type III plate was generated by a 0,2 mm thick and 30 mm wide polyethylene terephthalate (PET) foil strip (Kovács és Társa Kft., Budapest, Hungary) treated with Formula Five Mold Release wax laminated between the reinforcing layers. A [0/90^f^, *i*, ±45^f^, ±45^f^, 0/90^f^] stacking sequence was used, where *i* indicates the location of the PET foil. The summary of the produced samples can be found in Table 1.

**Figure 1:**
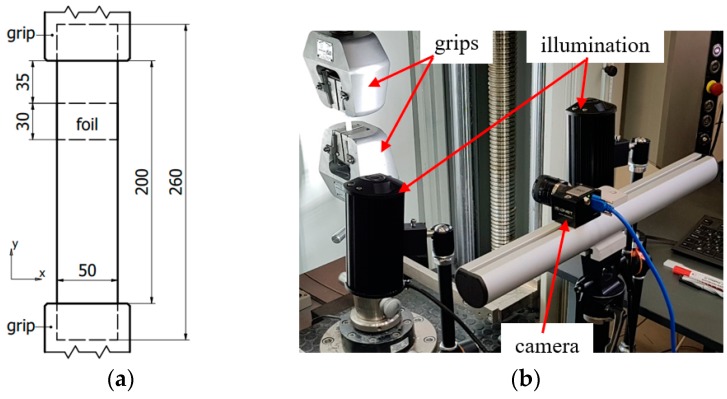
(**a**) Geometry of specimens; (**b**) the experimental setup.

The post-curing of the plates was performed in a Heraeus UT 20 (Heraeus, Hanau, Germany) drying oven at 60 °C for 4 h. Thereafter, 250 mm long, 25 mm wide specimens were prepared with a Mutronic Diadisc (Mutronic, Rieden am Forggensee, Germany) diamond disc cutter. Thereby through-failure was created in every specimen (Figure 1a). Finally, the black specimens exhibited a random surface pattern by white paint spray, which ensured sufficient contrast for the DIC investigation.

### 2.2. Visual Inspection (VI)

In the type I. specimens, the position of the white PTFE foil was visible at the cut edges, but all the layers moved together and the delamination did not open up by manual bending. In the type II. specimens, the position of the imperfection was not visible on the cut edges and there was also no buckling phenomenon for manual bending. In the type III. specimens, the PET foil was clearly visible on the cut edges. Due to the smaller stiffness of the plate and the poor adhesion, the layers did not move together in the target area, and the buckling phenomenon appeared at low load.

The surfaces of the specimens had different surface roughness because one side of the plate was in contact with a glass plate and the other side was formed by the peel ply release fabric. This had an effect on data analysis, which will be described in the discussion.

### 2.3. Mechanical Tests

Tensile tests were conducted at room temperature with a Zwick Z250 universal material testing machine equipped with a 250 KN load cell. Tests were performed with displacement control, loading rate was 2 mm/min. The strain limit was 1%, which was computed from crosshead displacement. The free length of the specimens was 200 mm. The experimental setup (Figure 1b) consisted of the following: a 5 Mpixel camera (MCR150-S, IDS GmbH., Obersulm, Germany) mounted on a tripod, two LED reflectors and a computer with Mercury RT software. The camera had a 25 mm lens, which was 300 mm from the surface and collected the data at 10 Hz, which proved to be adequate based on the first experiments with the used test speed. Increasing the number of acquired images does not improve the description of the phenomena, but unnecessarily increases the data size/computing time requirements. Before the test, the lens system was calibrated. There are two levels of camera calibration included in the software. The first level of calibration was used to remove the effect of distortions of the lens, which would otherwise negatively affect the results of the measurement. This was carried out by taking pictures of a well-defined calibrating grid from different angles. The second level of calibration defines the alignment of the coordinate system and the pixel to mm ratio. After calibration, the resulting resolution was 0.194 mm/pixel. It is worth noting that thanks to the tracked patterned window, the system is capable of subpixel measurement, so the accuracy can theoretically go below the optical resolution.

Among others the DIC method is very sensitive to the illumination and optical parameters and the available accuracy depends highly on the accuracy of the image which depends on the camera system and its settings. The used software is complex; it can help to control and optimize the camera and illumination parameters. The system was set up for the measurements as follows: Firstly, we fixed the aperture size in f/5.4 which resulted in an appropriate depth of field and enough light reaching the CCD sensor to have an adequately detailed picture. Then the illumination was turned on and we coarsely focused on the sample. The shutter time was set firstly with the software’s autoshutter tool, and tweaked manually afterwards. Then, with the help of the clippings and histogram tool, the position of illumination was modified to avoid the overexposed and underexposed areas of the image which cause loss of information and any projected shadows, which could mask the pattern. Thereafter, the focus distance was set by the help of the focus tool which visually indicates the best focal length by illuminating the areas, where the pattern is identifiable by good contrast and sharp contours. This tool also evaluates the quality of the surface pattern.

The compression tests were conducted with the same setup as previously described. The loading rate was 2 mm/min and the displacement limit was 3%, which was computed from crosshead displacement. The same specimens which were used during the tensile tests were reused for the compression tests in order to compare which test is more efficient to detect the artificial delamination. The fact that a low load was used for the non-destructive testing made it possible to conduct both tests on the same specimen, without damaging it.

## 3. Results and Discussion

The images were collected and analyzed by Mercury RT-v2.6 software run on a computer with an Intel Core i7-7700K processor, 8 GB of RAM and a Patriot Hellfire 480GB M.2 SSD. After the tests, we tried three virtual tools to find the best way of processing data. In all cases, 40 × 40 pixel windows, 0.2 confidence interval, high correlation quality, fast speed and full affine transformation were used. 

The default value of 0.2 confidence interval means that the found point is located within ±0.2 px within the computed location with the 95% probability. Correlation quality specifies the interpolation method used in correlation. More complex methods are slower. Fast speed means the method searches in the intermediate neighborhood (Normal speed searches in the whole image). The affine transformation type contains all three types together (translate, scale and shear).

According to our theory, the part of the specimen where the artificial delamination was placed (target zone) is going to deform differently than the intact surface (reference zone) due to the load because of the altered interaction between the adjacent layers. The reference and the target zone are of equal size and both symmetrical to the centerline of the specimen along the x axis.

### 3.1. Line Probes (LP)

**Figure 2:**
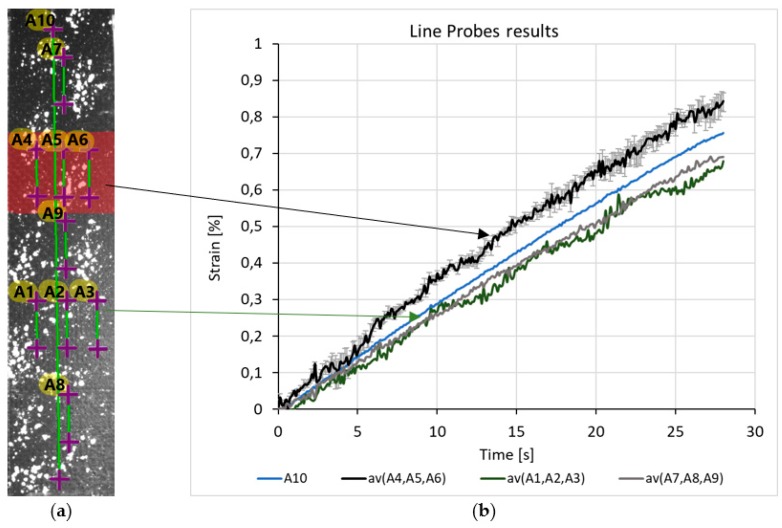
(**a**) Location of line probes in the first specimen (**b**) strain-frame diagram from line probes.

Ten pieces of 20 mm long, virtual LP-s, parallel to axis y were placed on the surface of the specimen: 3 pieces on the reference zone (A1, A2, A3), 3 pieces on the target zone (A4, A5, A6), 3 pieces on the other intact parts of the specimen and 1 piece of 175 mm long LP, which measured the global strain of the specimen (A10) (Figure 2a). It can be observed that the 1% strain calculated from the displacement of the crosshead, which controls the tensile machine, resulted only in 0.75% strain. The rest was the result of the deformation of the machine frame and the movement of the grip. Despite the fact that these LP-s show similar results in (%) strain, if one wants to detect the location of the artificial fatigue, the smallest differences have to be analyzed as well.

Firstly, it can be seen that the strain curve of A10 LP is smoother, due to averaging the stochastic local changes. Secondly, the A10 curve divides the diagram into two significantly different parts, with which it displays the strains of the zones. Above the A10 curve there is the av(A4, A5, A6), which is the average (av) of the target zone’s LPs, showing the scatter as well. The reference curves, av(A1, A2, A3) and av(A7, A8, A9) are under the A10 curve. These curves are also average values, but the scatters are not shown because they overlap.

To sum up, during the tensile test, larger local, longitudinal strains were detected in the target zone by LP-s, which indicates the existence and the approximate location of the artificial delamination. To put it in numbers, with 0,4% strain of the specimen (based on A10) the strain of target zone is 19% higher than the strain of the reference zone. 

### 3.2. Strain Gauges (SG)

**Figure 3:**
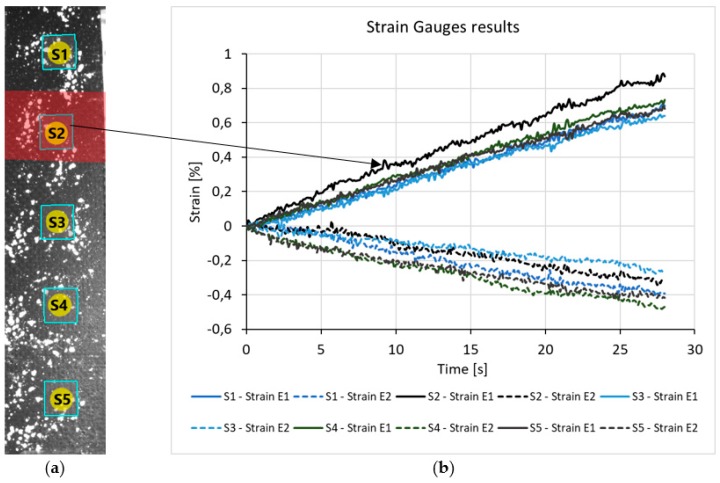
(**a**) Location of strain gauges in the first specimen (**b**) strain-frame diagram from virtual strain gauges.

Another tool for strain measurement with Mercury RT is a virtual strain gauge, which can be imagined as a square. The corner points are followed by DIC, while longitudinal and transverse strains are computed along the edges. Five pieces of virtual SG-s were placed on the surface of the specimens (S1, S2, S3, S4, S5), this is shown by Figure 3a. The S2 was located at the target zones of every specimen. Similar to LP-s, different longitudinal strain was detected by S2 compared to the other SG-s (Figure 3b). Contrary to LP-s, in this case the transverse strains were also obtained, but these did not show significant difference between reference and target areas. However, the transverse strain might be important, when the material, the load, or the geometry of the imperfection differs, for example: embedded delamination or compression load. Therefore, instead of using LP-s it is advisable to choose SG-s since we get more useful data in the same amount of computation time (2 s). 

### 3.3. Full Field Strain Measurement

The third method for evaluation is collecting data with full field measurement. In this case, the region of interest (ROI) is selected, in which the number of points can be chosen. The more points there are, the more accurate the results will be, but the computation time will be longer. A 10-pixel grid space was used in the ROI, which resulted in about 1500 points. The computation times were about 1 minute for each specimen.

**Figure 4:**
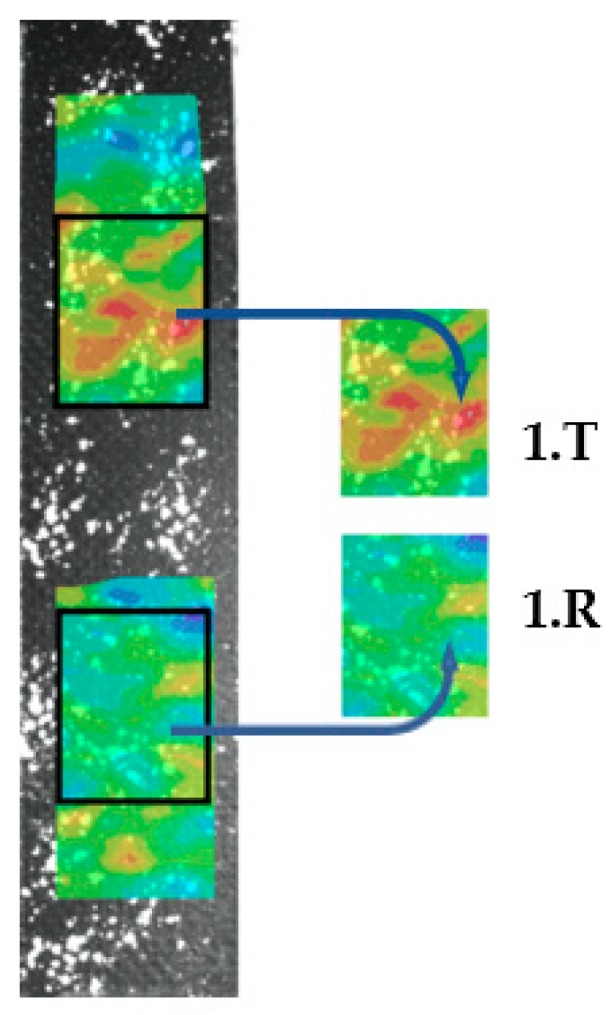
Interpretation of cut out surface strain fields.

For every evaluated property, there is a colored map, which can be viewed on a global (referring to the whole test) or a local (referring to the actual image) scale. By choosing the global scale, only the main changes can be tracked. This means that the effect of the fault is significantly smaller, so a search for the local outliers is needed. The result is presented at 0,4% average strain because in most cases, outlier values appeared on the surface of the specimens. The target and the reference zones were cut and compared to each other at same fixed scale in the 0.15–0.65% strain range, as shown in Figure 4. T denotes the target zone, which contains the delamination, R denotes the reference zone, the numbers before the letters denote the number of specimens. The position of R and T change in a table, because in some cases the specimen was rotated by 180° in order to show that the phenomenon is not the result of specimen gripping. This way, all of the specimen’s full deformation fields can be compared to each other (Figure 4.).

Note 1: During the whole test, the outliers slightly fluctuated but did not disappear. 

Note 2: In order to reduce computation time, when the tensile test was processed, instead of a full specimen surface analysis, it is an option to eliminate certain areas and analyze only the target and the reference zones. Sadly, this simplification was not applicable for the compression test, due to the buckling phenomenon.

**Figure 5:**
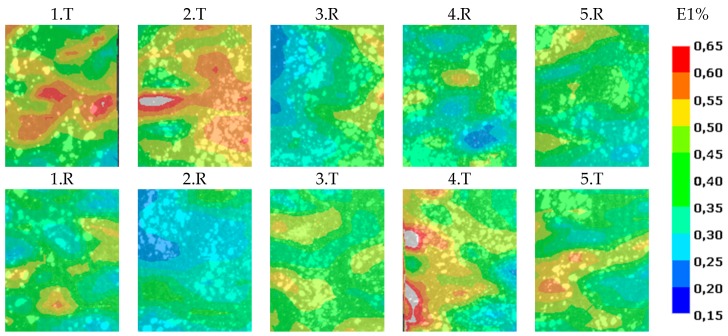
Comparison of longitudinal strains of specimens 1–5. during the tensile test at the 0.4% average strain condition.

As shown in Figure 4 or Figure 5, the target zones contain outlier strain values (orange and red areas) which identify clearly the delaminations. The target zones were located on the upper part of specimens 1. and 2. when the target zones were located on the lower part of specimens 3–5. The target areas produced consistently higher strains so the outlier phenomenon is not related to the delamination located at the top or bottom.

**Figure 6:**
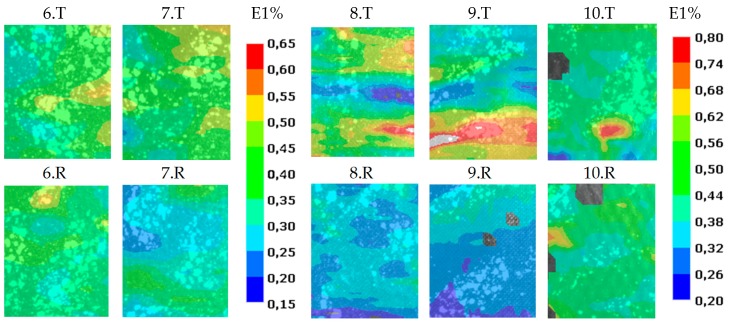
Comparison of longitudinal strains of specimens 6–10. during the tensile test.

No significant difference was found between target and reference zones in the case of specimens 6. and 7. (Figure 6), so we conclude that artificial delamination was not created by only wax in the type II specimens.

Specimens 8. and 9. showed a well separated, striped pattern (Figure 6). In this case the foil was behind the examined top layer. When specimen 10. was tested, the specimen was rotated by 180° around the long side, so the delamination was further away from the examined surface. There was no striped pattern, just a small red speck appeared when it was analyzed by the DIC method. This means increasing the number of layers between the camera and delamination can decease or hide the effect of a fault.

**Figure 7:**
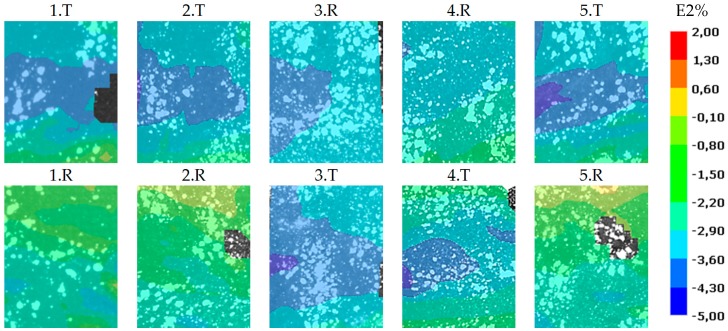
Comparison of transverse strains of specimens 1–5. during the compression test at −1.5% average strain.

Figure 7. shows the results of the compression tests of type I. specimens. The result is presented at −1.5% average transverse strain. The target and the reference zones were cut and compared to each other with same fixed scale in the range between −5–2% compressive strain. This result confirms the results of the tensile tests. Where the strain map showed outlier longitudinal values at the tensile test, there the strain map showed outlier transversal strain values at the compression test.

Note 3: The specimens buckled out of plane at compression tests, and we used 2D DIC so the measured values deviate from the real values. These are just a projections of 3D alterations. However, it is not crucial in this case, because we just want to detect the delaminations, and the 2D DIC method was found suitable for this task.

The type II. specimens did not show significant difference between target and reference areas in case of compression tests too. The examined surface of type III. specimens was not matte enough because these were in contact with a glass plate during the production. When these specimens buckled as the load increased, the surface reflected the light of the illumination, thus the following of the pattern by DIC failed gradually.

It is not sufficient to have a random patterned surface with good contrast, it needs to be matte too, which can be achieved by the application of a primer paint. If it is not possible, polar filters have to be used.

## 4. Conclusions

There are many non-destructive tests used by the industry for detecting imperfections including, but not limited to, computer tomography, infrared thermography, acoustic emission method and ultrasound test but these all have both advantages and disadvantages. Therefore, investigation and development of other nondestructive tests are important to be able to be able to perform ND tests in different applications. The DIC method combined with small loads is a promising method for the detection of faults because it is a fast, non-contact test. Moreover, there are no material limitations and it can be used on large or hot and cold surfaces too. At present, some non-destructive methods are able to give more accurate results about location and geometry of delamination than the DIC method, but these methods can be complicated and slow. In a few years the DIC method could be applied at the end of a production line for inspection or as a quality control, or it could be included in the automatic revision of the wings of airplanes in the hangar. In our opinion this could be a quick comprehensive examination to search for suspicious places which would be re-examined by a more accurate and more time-consuming technology.

In this work, carbon or glass fabric-reinforced composites were tested by low load level tensile and compression test while the surfaces of specimens were investigated and then analyzed by the 2D DIC method. The specimens contained an artificial through-delamination which was detected from a strain map by DIC in most cases. The delaminations were created by foils during production. The delamination in the carbon fiber reinforced specimens remained closed before tests, in contrast, in the glass reinforced specimens the delaminations could be opened before the tensile test. These showed different pattern in the surface strain map of the delaminated zone, but the phenomenon was typical of the type of specimen.

When tensile tests were performed we detected higher longitudinal strain values in the delaminated zone than in the intact reference zone. In contrast, when compression tests were performed we detected higher absolute transversal strain values in the delaminated zone than in the intact reference zone. We checked that the strain differences were not caused by the specimen gripping.

Three methods were applied for evaluating the strain of which the full-field measurement was the main direction. However, it required longer computation than virtual line probes and strain gauges, but it is necessary when we do not know the location of delamination.

It was also shown that increasing the number of layers between the camera and delamination can hide or decease the effect of the fault. This masking effect is a very important question of the application, so we are going to examine it in the future. Moreover, we are going to investigate the effect of delamination geometry, which will be validated by FEM.

## Figures and Tables

**Table 1:** Sample classifications, compositions and results summary.

Specimen Type	I.					II.		III.		
**material**	200 g/m^2^ carbon fabric							220 g/m^2^ glass fabric		
**stacking sequence**	[0/90^f^, ±45^f^, *i*, ±45^f^, 0/90^f^]							[0/90^f^, *i*, ±45^f^, ±45^f^, 0/90^f^]		
**delaminated by**	PTFE foil					just wax without foil		PET foil+ wax		
**geometry of delamination**	30 mm wide, through-delamination									
**specimen No.**	1	2	3	4	5	6	7	8	9	10
**position of delamination between grips**	up	up	down (rotated by 180°)	down	down	up	up	up	up	up (behind)
**delamination is detected by VI**	no	no	no	no	no	no	no	yes	yes	yes
**delamination is detected by DIC**	yes	yes	yes	yes	yes	no	no	yes	yes	yes
